# Outcome of Corneal Collagen Crosslinking for Progressive Keratoconus in Paediatric Patients

**DOI:** 10.1155/2014/140461

**Published:** 2014-06-11

**Authors:** Deepa Viswanathan, Nikhil L. Kumar, John J. Males

**Affiliations:** ^1^Australian School of Advanced Medicine, Macquarie University, Sydney, NSW 2109, Australia; ^2^Sydney Adventist Hospital Clinical School, The University of Sydney, Sydney, NSW 2076, Australia; ^3^Sydney Eye Hospital, The University of Sydney, Sydney, NSW 2000, Australia

## Abstract

*Purpose*. To evaluate the efficacy of corneal collagen crosslinking for progressive keratoconus in paediatric patients. *Methods*. This prospective study included 25 eyes of 18 patients (aged 18 years or younger) who underwent collagen crosslinking performed using riboflavin and ultraviolet-A irradiation (370 nm, 3 mW/cm^2^, 30 min). *Results*. The mean patient age was 14.3 ± 2.4 years (range 8–17) and mean followup duration was 20.1 ± 14.25 months (range 6–48). Crosslinked eyes demonstrated a significant reduction of keratometry values. The mean baseline simulated keratometry values were 46.34 dioptres (D) in the flattest meridian and 50.06 D in the steepest meridian. At 20 months after crosslinking, the values were 45.67 D (*P* = 0.03) and 49.34 D (*P* = 0.005), respectively. The best spectacle corrected visual acuity (BSCVA) and topometric astigmatism improved after crosslinking. Mean logarithm of the minimum angle of resolution (logMAR) BSCVA decreased from 0.24 to 0.21 (*P* = 0.89) and topometric astigmatism reduced from mean 3.50 D to 3.25 D (*P* = 0.51). *Conclusions*. Collagen crosslinking using riboflavin and ultraviolet-A is an effective treatment option for progressive keratoconus in paediatric patients. Crosslinking stabilises the condition and, thus, reduces the need for corneal grafting in these young patients.

## 1. Introduction

Keratoconus is a degenerative corneal disorder charecterised by corneal thinning, conical protrusion, irregular astigmatism, and visual impairment [1]. Keratoconic eyes have an altered corneal biomechanical profile and appear to be more elastic and less rigid than normal eyes [2]. Keratoconus usually manifests during adolescence and early adulthood. Young patients are at risk for faster disease progression and corneal grafting often becomes necessary for visual rehabilitation [3].

Corneal collagen crosslinking (CXL) is a recently introduced treatment for addressing progressive keratoconus. It is a minimally invasive procedure and the only option that halts or slows disease progression. Riboflavin and ultraviolet-A induce crosslinking through photopolymerization of collagen mediated by reactive oxygen species and, thus, increase corneal biomechanical rigidity and biochemical resistance [4–6].

Several clinical studies have demonstrated that CXL effectively slows keratoconus progression in adult eyes [7–13]. Recently, CXL has been recommended as an optimal intervention for progressive Keratoconus affecting the paediatric population [14–17].

Therefore CXL could potentially reduce the need for corneal grafting in these young individuals. This is particularly relevant as paediatric patients have a greater risk of corneal transplant rejection [17]. We observed favourable results after CXL in adult eyes [13] and this study aims to evaluate its efficacy in treating progressive keratoconus affecting paediatric subjects.

## 2. Materials and Methods

Twenty five eyes of 18 patients (5 females, 13 males) with progressive keratoconus underwent CXL and were enrolled in this prospective study. Only patients who completed a minimum of 6 months follow-up after the procedure were included. The institutional ethics committee approved the study and parents provided informed consent prior to treatment.

### 2.1. Inclusion Criteria

Patients aged less than 18 years with progressive early to moderate keratoconus (grades I to III according to the Amsler-Krumeich classification) with a minimum corneal thickness of at least 400 microns were included [18]. Indications for treatment included an increase in steep keratometry of 1.00 dioptre (D) or more in 1 year, deterioration in visual acuity, and the need for new contact lens fitting more than once in 2 years. Exclusion criteria were advanced keratoconus with stromal scarring, corneal thickness less than 400 microns, corneal hydrops, severe dry eye, corneal infections, previous ocular surgery, and autoimmune diseases.

### 2.2. Tests and Evaluation

Soft contact lenses were discontinued for a minimum of 3 days and rigid-gas permeable and hard lenses were discontinued for minimum of 2 weeks before preoperative eye examination. Evaluation of visual acuity, manifest refraction, corneal topography, and corneal pachymetry was performed preoperatively and postoperatively in all subjects. The logMAR BSCVA was obtained using the early treatment of diabetic retinopathy study chart (ETDRS). Manifest refraction was performed and the manifest refraction spherical equivalent (MRSE) was analysed. Corneal topography and corneal thickness measurements (pachymetry) were performed using a noncontact rotating Scheimpflug camera (Pentacam, Oculus Inc., Germany).

### 2.3. Crosslinking Technique

Corneal collagen crosslinking was performed using 0.1% riboflavin (in 20% dextran T 500) and ultraviolet A (UVA) irradiation (370 nm, 3 mW/cm^2^, 30 min) under sterile conditions. The UV-X 1000 machine (IROC Innocross AG, Zurich, Switzerland) and the Innocross-R riboflavin isotonic solution (riboflavin 5-phosphate (0.1%) plus 20% Dextran T500 in 2 mL syringes) were used. The procedure was performed under general anaesthesia in very young patients and under topical anaesthesia in older patients. After anaesthesia, a lid speculum was inserted and the corneal epithelium was soaked with 20% alcohol for 40 seconds. The epithelial tissue was then removed in a 9.0 mm diameter area with a cellulose surgical spear to allow penetration of riboflavin into the corneal stroma. Thereafter, the photosensitizer 0.1% riboflavin was applied (2 to 3 drops every 3 minutes) to the cornea for 30 minutes before irradiation to allow sufficient saturation of the stroma.

Corneal soaking of riboflavin was assessed and then the central 8.0 mm cornea was exposed to UVA light (wavelength of 370 nm and irradiance of 3 mW/cm^2^) for 30 minutes. Throughout the UVA exposure, riboflavin solution was instilled (2 to 3 drops every 3 minutes). Upon completion of treatment, the eye was washed with balanced salt solution and antibiotic eye drops (ofloxacin 0.3%) and steroid eye drops (dexamethasone 0.1%) were applied. A bandage contact lens was placed in the eye until complete reepithelialization. Subsequent follow-up examinations were performed at 1 week and thereafter at 1, 6, 12, 18, and 24 months and annually thereafter. The BSCVA, corneal topography, and central corneal thickness (CCT) were recorded at each visit.

### 2.4. Statistical Analysis

The changes in simulated keratometry values in the flattest meridian (*K1*) and the steepest meridian (*K2*), topometric astigmatism, manifest refraction, and BSCVA were analysed to evaluate the effect of crosslinking treatment. This was performed by subtracting each parameter at the respective follow-up examination from the preprocedure value. Postprocedure data was available for all 25 eyes. Statistical evaluation was performed by SPSS software version 19. The paired *t*-test was used to evaluate the differences in the different parameters between pre- and postprocedure values and a *P* value of ≤0.05 was considered to be statistically significant.

## 3. Results

The mean patient age was 14.3 ± 2.4 years (range 8–17 years); there were 5 females and 13 males. The risk factors for Keratoconus development in the patient population included eye rubbing in 58.8% patients and atopy in 47.10% patients. The outcomes after crosslinking at mean follow-up of 20.1 ± 14.25 months (range 6–48 months) are shown in Table 1.

*Visual Acuity.* The mean logMAR BSCVA improved by 0.02 ± 0.19 (*P* = 0.89) at mean 20-month follow-up after CXL.

*Manifest Refraction.* There was a reduction in mean spherical equivalent from −5.66 ± 3.47 D to −4.71 ± 3.11 D (*P* = 0.71) in treated eyes at mean follow-up of 20 months.

*Corneal Topography.* There was a significant reduction in keratometry values following crosslinking. The mean simulated keratometry value in the flattest meridian (*K1*) reduced by 0.66 ± 1.38 D (*P* = 0.03) and the mean simulated keratometry value in the steepest meridian (*K2*) reduced by 0.72 ± 1.17 D (*P* = 0.009) at 20-months follow-up.

**Figure 1:**
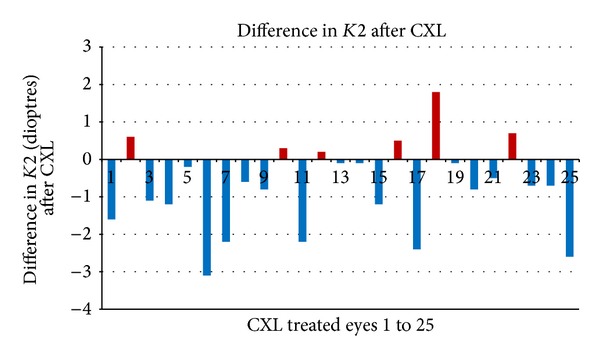
Difference in* K2* between pre- and post-CXL treated eyes.

There was a decrease in topometric astigmatism by 0.20 ± 1.44D (*P* = 0.51) after crosslinking.  Figure 1 shows the difference in* K2* between pre- and post-CXL treated eyes at mean 20-month follow-up. Corneal curvature was either reduced or remained stable (within 0.5 D of pre-CXL* K2*) after CXL in 88% (22/25) eyes.

**Figure 2:**
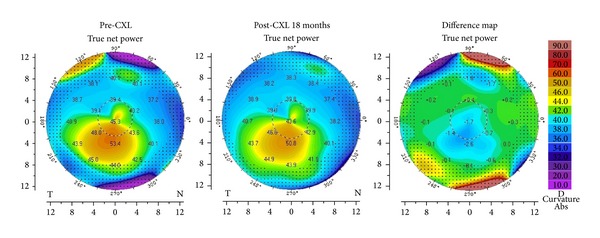
Corneal topography showing a reduction in keratometry after CXL.

Corneal topography of a crosslinked eye is shown in Figure 2. At 18 months after CXL, there was a reduction in* K2* by 1.8 D in the treated eye. No serious complications like infections or stromal scarring were noted in this series.

## 4. Discussion

We assessed the topographic, refractive, and visual outcomes of corneal UV collagen crosslinking in a cohort of paediatric patients with progressive keratoconus. At 20 months after crosslinking, there was a mean reduction in simulated keratometry values by 0.66 D in the flattest meridian and by 0.72 D in the steepest meridian. This was associated with an improvement in visual acuity and topometric astigmatism, although these improvements were not statistically significant.

Collagen crosslinking involves a photopolymerization reaction that induces biochemical and microstructural changes within the corneal stroma [4–6]. These include the generation of stiffer collagen fibrils and a rearrangement of corneal lamellae within the matrix [19, 20]. These structural and biomechanical changes after crosslinking result in a regression of corneal curvature and improved shape thus stabilising keratoconus and preventing further progression.

We had previously reported favourable results after collagen crosslinking in adult Keratoconic eyes consistent with other published studies [8–13]. Similar to our adult cohort, this study included only patients who had completed a minimum of 6 months follow-up after CXL. This is based on previous long term studies that reported an initial worsening of corneal curvature followed by subsequent flattening and stabilisation after CXL [9, 11].

In recent times, the age limit for CXL has lowered considerably [14–16]. In this study, the youngest patient was 8 years old and to the best of our knowledge is the youngest patient reported to undergo crosslinking. Vinciguerra et al. evaluated the long term outcome of CXL for progressive keratoconus in different age groups, including 49 eyes of patients aged below 18 years [21]. Interestingly, their results indicated better functional and morphologic outcomes in young adults (age 18–39 years) as compared to the paediatric age group.

Arora et al. conducted a prospective contralateral case control study and included 15 eyes of 15 keratoconic patients that underwent CXL [17]. The criteria for performing CXL were not documented progressions, but the advanced keratoconus status in the fellow eye. At 1 year after CXL, significant improvements were noted in logMAR BSCVA and apical keratometry. In comparison, CXL was performed only on progressive paediatric keratoconic eyes in the current study and albeit a longer follow-up, significant improvements were noted only for keratometry values and not for BSCVA.

Magli et al. recently compared the efficacy of transepithelial CXL (TE-CXL) to conventional epithelium-off CXL in paediatric patients [22]. At 12-month follow-up, they observed that TE-CXL had similar efficacy, but was less painful and had fewer complications than epithelium-off CXL. Similarly, Salman performed a prospective case control study on the efficacy and safety of TE-CXL in children and reported satisfactory results [23]. In the current study, crosslinking was performed using the epithelium-off technique and no serious complications were noted.

In their series of paediatric crosslinking, Caporossi et al. report worsening in terms of topographic and pachymetic data in 4.6% of eyes; however the term “worsening” is not defined [16]. We observed worsening of the steep keratometry value (*K2*) by more than 0.5 D in 3 crosslinked eyes. However, an increase in* K2* exceeding 1 D occurred in only 1 eye which was not associated with a decrease in BSCVA. We presume that this reflects the fast rate of keratoconus progression in paediatric eyes. Therefore, earlier studies have suggested a closer follow-up schedule for children with keratoconus to rapidly identify deterioration.

This study demonstrates that collagen crosslinking can result in a significant reduction in corneal curvature and can stabilise progressive keratoconus in patients younger than 18 years. These encouraging results emphasize the need for early treatment in these young patients to prevent them from unnecessarily undergoing corneal grafting. The optimal timing of intervention however remains debatable with some authors suggesting crosslinking at diagnosis of keratoconus without awaiting disease progression [24].

## Figures and Tables

**Table 1:** Pre- and postcrosslinking data for treated eyes.

Parameter	Pre-CXL	Post-CXL	*P* value
BSCVA (logMAR)	0.24 ± 0.19	0.21 ± 0.13	0.89
MRSE (dioptres)	−5.66 ± 3.47	−4.71 ± 3.11 D	0.71
*K*1 (dioptres)	46.34 ± 3.13 D	45.67 ± 3.31 D	0.03
*K*2 (dioptres)	50.06 ± 3.84 D	49.34 ± 3.18 D	0.005
Topometric astigmatism (dioptres)	3.50 ± 1.36 D	3.25 ± 1.79 D	0.51

BSCVA: best spectacle corrected visual acuity, logMAR: logarithm of the minimum angle of resolution, MRSE: manifest refraction spherical equivalent, *K*1: mean simulated keratometry value in the flattest meridian, and *K*2: mean simulated keratometry value in the steepest meridian.
